# NRG Oncology Liver Proton SBRT and Hypofractionated Radiation Therapy: Current Treatment Technical Assessment and Practice Patterns

**DOI:** 10.3390/cancers17142369

**Published:** 2025-07-17

**Authors:** Minglei Kang, Paige A. Taylor, Jiajian Shen, Jun Zhou, Jatinder Saini, Theodore S. Hong, Kristin Higgins, Wei Liu, Ying Xiao, Charles B. Simone, Liyong Lin

**Affiliations:** 1 New York Proton Center, New York, NY 10035, USA; csimone@nyproton.com; 2 Department of Human Oncology, University of Wisconsin, Madison, WI 53792, USA; 3 Imaging and Radiation Oncology Core Houston Quality Assurance Center, University of Texas MD Anderson Cancer Center, Houston, TX 77030, USA; pataylor@mdanderson.org; 4 Department of Radiation Oncology, Mayo Clinic, Phoenix, AZ 85054, USA; shen.jiajian@mayo.edu (J.S.); liu.wei@mayo.edu (W.L.); 5 Department of Radiation Oncology and Winship Cancer Institute, Emory University, Atlanta, GA 30322, USA; jun.zhou@emory.edu (J.Z.); liyong.lin@emoryhealthcare.org (L.L.); 6 Department of Radiation Oncology, University of Washington School of Medicine, Seattle, WA 98133, USA; jatinder.saini@seattleprotons.org; 7 Radiation Oncology, Massachusetts General Hospital and Harvard Medical School, Boston, MA 02114, USA; tshong1@mgh.harvard.edu; 8 Department of Radiation Oncology, City of Hope Atlanta, Newnan, GA 30265, USA; kristin.higgins@coh.org; 9 Department of Radiation Oncology, University of Pennsylvania, Philadelphia, PA 19104, USA; ying.xiao@pennmedicine.upenn.edu

**Keywords:** proton therapy, liver, stereotactic body radiation therapy, hypofractionated therapy, image-guided radiation therapy

## Abstract

**Background/Objectives:** Proton therapy delivers highly conformal doses to the target area without producing an exit dose, minimizing cumulative doses to healthy liver tissue. This study aims to evaluate current practices, challenges, and variations in the implementation of proton stereotactic body radiation therapy (SBRT) and hypofractionated therapy for liver malignancies, with the goal of providing a technical assessment to promote broader adoption and support future clinical trials. **Methods and Materials**: An extensive survey was conducted by NRG Oncology across North American proton treatment centers to assess the current practices of proton liver SBRT and hypofractionated therapy. The survey focused on key aspects, including patient selection, prescription and normal tissue constraints, simulation and motion management, treatment planning, quality assurance (QA), treatment delivery, and the use of image-guided radiation therapy (IGRT). **Results:** This survey captures the current practice patterns and status of proton SBRT and hypofractionated therapy in liver cancer treatment.  Proton therapy is increasingly preferred for treating inoperable liver malignancies due to its ability to minimize healthy tissue exposure. However, the precision required for proton therapy presents challenges, particularly in managing uncertainties and target motion during high-dose fractions and short treatment courses. Survey findings revealed significant variability in clinical practices across centers, highlighting differences in motion management, dose fractionation schedules, and QA protocols. **Conclusion:** Proton SBRT and hypofractionated therapy offer significant potential for treating liver malignancies. A comprehensive approach involving precise patient selection, treatment planning, and QA is essential for ensuring safety and effectiveness. This survey provides valuable insights into current practices and challenges, offering a foundation for technical recommendations to optimize the use of proton therapy and guide future clinical trials.

## Simple Summary

Proton therapy can deliver radiation very precisely to tumors, helping to better protect healthy tissue. This makes it a promising option for treating liver cancers that cannot be removed by surgery. Proton stereotactic body radiation therapy (SBRT) and hypofractionated therapy are increasingly used in these cases. However, proton therapy requires a high level of accuracy, which can be challenging—especially when treating tumors that move with breathing or when using high doses over fewer sessions. To ensure safe and effective treatment, several steps must be carefully managed, including patient selection, setting dose limits, managing motion, treatment planning, quality assurance, and image guidance. Since different centers may use different approaches, a detailed survey was conducted to better understand how proton therapy is currently used for liver cancer across North America. The goal is to identify current practices and challenges, and to support broader use of this therapy and future clinical trials, with guidance from NRG Oncology.

## 1. Introduction

Radiation therapy (RT) is an essential option for treating inoperable liver cancers. The liver is a parallel organ, and the toxicity of radiation-induced liver disease (RILD) is mainly associated with integrated doses to the normal liver tissue [1]. Stereotactic body radiation therapy (SBRT) and hypofractionated radiation therapy usually treat small targets with highly focal dose deposition, which has the advantage of reducing mean doses to normal liver tissues. Advancements in various techniques, such as imaging, treatment planning, motion management, and delivery, have made it possible to administer treatments in fewer fractions with highly localized and precise delivery. These improvements in planning and therapy techniques have significantly increased the adoption of high-dose SBRT and hypofractionation in the treatment of both hepatocellular carcinoma (HCC) and liver metastases.

Numerous studies have reported using SBRT to treat liver metastases [2,3,4,5,6,7]. A multi-institutional phase I/II study of SBRT for the liver with one to three hepatic metastases showed that it is safe to escalate the dose from 36 up to 60 Gy in 3 fractions, with a 2-year local control rate of 92% [4]. Goodman et al. reported using SBRT to treat hepatic metastases, showing a high local control rate of >90% [5]. In HCC treatment, a single-arm phase II study showed that SBRT is safe and effective, with a 1-year LC rate above 90% [6]. A retrospective study conducted by Ohri et al. also confirmed the effectiveness of SBRT in treating primary and metastatic liver tumors [8]. The study found that higher doses were associated with better local control rates. This indicates that using SBRT, which allows for precise radiation targeting, can improve outcomes in treating liver tumors when higher radiation doses are administered while minimizing exposure to surrounding healthy tissues [8]. The use of SBRT and hypofractionation in treating liver cancer continually advances, particularly in determining the optimal dose, fractionation schedule, and techniques. The effectiveness of these treatments is closely linked to the size of the fractionation. For tumors located near critical gastrointestinal organs, a risk-adapted approach with an increased number of fractions is often advisable to minimize potential complications [9].

In recent years, the use of proton therapy has increased rapidly. Compared to photon therapy, proton therapy offers a more appealing approach to treating liver cancers [10]. The unique characteristics of Bragg peaks are that they can release most energy within a few millimeters, resulting in almost zero exit dose beyond the target. Proton therapy allows for the precise targeting of tumors while minimizing damage to surrounding healthy tissues. The organs-at-risk (OARs) distal to the tumor receive almost no dose. Proton pencil beam scanning (PBS) is the latest format of proton radiation therapy modality that offers superior dose conformity and high linear energy transfer (LET) compared to conventional photon and electron RT [11]. The ability of proton therapy to spare normal tissues beyond the target renders it particularly beneficial for treating liver cancers, where RILD from high irradiation doses to liver tissues can affect the quality of life and survival of these patients. This characteristic significantly reduces the integral doses to the surrounding normal liver tissues, minimizing potential side effects and enhancing the safety of the treatment for sensitive areas.

The superior normal tissue protection of proton therapy allows tumor dose escalation [12,13,14,15,16] ablative radiation doses to treat liver tumors that have the potential to reduce toxicities and improve survival relative to photon therapy [17,18]. Prospective studies [19,20] and clinical trials have demonstrated that proton SBRT and hypofractionation can deliver safe, accurate, and efficacious treatment for liver cancers [21,22]. The dosimetry studies also show that protons can achieve better normal tissue sparing, potentially leading to fewer side effects and/or better quality of life than conventional photon radiation. Based on the previous clinical phases I and II trials [22] and population-based data favoring proton therapy over photon SBRT for hepatocellular cancer, the ongoing NRG Oncology GI003 phase III trial assessing proton versus photon therapy for hepatocellular carcinoma may more fully establish the role of proton SBRT and hypofractionation as a standard of care in this patient population [23].

In general, SBRT and hypofractionated therapy have been less extensively researched for liver cancer, particularly within proton therapy [21,22]. According to the live vote at NRG Oncology SBRT workshop debate between proton and photon arms in 2022, the majority of proton centers in the United States are ready to use proton SBRT and hypofractionated therapy for liver cancer. To navigate the complexities of proton SBRT and hypofractionated therapy for liver cancer treatments effectively, it is essential to capture their prevailing practice patterns and current status. In response to this need, the NRG Oncology Medical Physics Subcommittee, supported by the NRG Oncology Particle Therapy Working Group, formed a dedicated workgroup to examine the application of SBRT and hypofractionated proton radiation therapy for treating liver cancer. This workgroup included radiation oncologists and therapeutic medical physicists with expertise in proton therapy. In August 2021, they dispatched a comprehensive questionnaire to all 29 United States proton centers engaged in NRG Oncology clinical trials to gather insights into the current practices of proton SBRT and hypofractionated therapy for liver cancer. The findings from this survey are intended to provide clinical guidance and serve as a reference for new proton centers looking to implement liver proton SBRT and hypofractionation.

## 2. Methods and Materials

The NRG Oncology Medical Physics Subcommittee created the questionnaire on proton liver SBRT and hypofractionated therapy. It contained 66 questions spanning five key aspects: (1) vendors, delivery techniques, and treatment planning system (TPSs), (2) patient selection, (3) simulation and motion management, (4) treatment planning and quality assurance (QA), and (5) motion management and image-guided radiation therapy (IGRT). This research concentrated on the latest developments in proton therapy. It offers a detailed overview of the clinical procedures and methods for applying proton SBRT and hypofractionated therapy in liver cancer treatment. Additionally, the study highlights the immediate requirements and potential advancements that could enhance the clinical application of proton SBRT and hypofractionation for treating liver cancer.

## 3. Proton Therapy Using SBRT and Hypofractionation for Liver Cancer Treatment

### 3.1. Survey Response Overview

NRG Oncology Medical Physics Subcommittee on Proton Liver SBRT and hypofractionated therapy distributed the survey to 29 proton centers in the United States, and 19 effective survey results were received, with a response rate of 66%. Among the 19 proton centers that completed the survey, 18 had clinical practices in place for treating liver malignancies using proton SBRT and/or hypofractionation, and one proton center anticipated implementing proton SBRT and/or hypofractionation in the treatment of liver cancers in the near future. Table 1 lists the distributions of proton system vendors, beam delivery techniques, and TPSs that are clinically among all 18 proton centers delivering proton SBRT or hypofractionation. Thirteen of the 18 proton centers passed IROC (Imaging and Radiation Oncology Core) liver motion phantom credentialing. The majority of proton therapy center respondents, 14 out of 18, employ the PBS technique. Only 4 centers exclusively utilize passive scattering methods. Among the 14 centers using PBS, one center is also equipped with a uniform scanning technique that is used for liver proton SBRT and hypofractionated therapy.

### 3.2. Patient Selection

**Figure 1:**
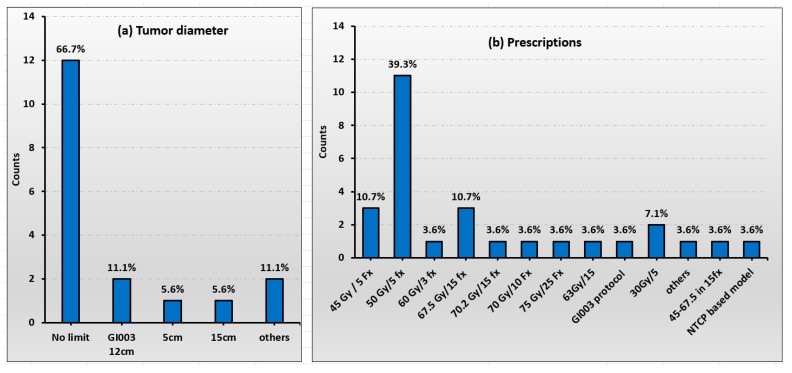
The statistics of the tumor diameter in (**a**) and the dose-fractionation regimes in (**b**) for proton SBRT and hypofractionated therapy.

Both primary liver tumors and liver metastases are treated among the 18 United States proton centers. Patients treated included non-surgical candidates or those who declined surgery for primary and metastatic liver tumors. Figure 1 illustrates the statistical data, with part (a) showing the tumor diameters and part (b) presenting the prescribed treatments. Patient selection criteria included patients with 1–3 liver metastases (18 out of 18), and the uninvolved liver volume should be >700 cc (16 out of 18). The survey reveals that 12 out of 18 centers do not define a specific individual tumor diameter for treatment. However, 1 out of 18 centers will treat tumors with a diameter of less than 5 cm, while 2 out of 18 centers treat those with diameters under 15 cm. Similarly, another 2 out of 18 centers apply SBRT/hypofractionation for tumors less than 15 cm in diameter. The most common SBRT protocol used is 50 GyRBE delivered in 5 fractions. Other frequently used hypofraction regimens include 45 Gy in 5 fractions and 60 Gy in 3 fractions. A wide range of fractionation schemes is also evident, as shown in Figure 1b.

### 3.3. Simulation and Motion Management

**Figure 2:**
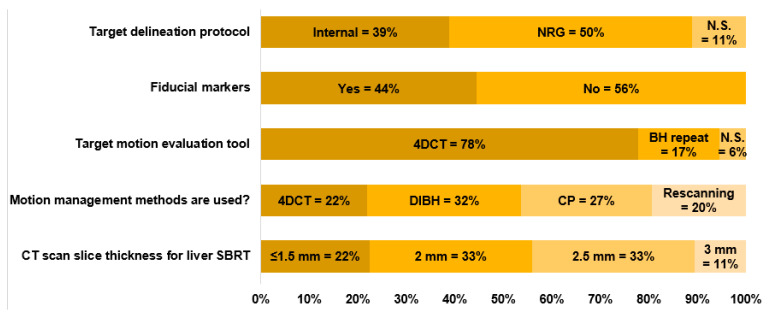
Target delineation protocols, utilization of fiducial markers, target motion evaluation, and CT slice thickness choice.

The vacuum cushion is the most prevalent for immobilization, used by 9 out of 18 centers. The combination of wing board and vacuum cushion ranks as the second most common approach, adopted by 4 out of 18 centers. Additionally, four centers utilize a pod and foam cushion setup, and one center employs a body mask for immobilization. As shown in Figure 2, approximately 44% of the 18 centers used fiducial markers to locate the tumor, whereas the remaining 56% did not use any markers for tumor localization or visualization. Around 50% of the centers used the NRG Oncology GI003 protocol to delineate the target, 39% used internally defined protocols, and the remaining 11% did not specify any protocols.

CT slice thickness plays a crucial role in ensuring the precision of target delineation, managing image data size, and maintaining dose accuracy. Among the centers surveyed, 2 mm and 2.5 mm slice thicknesses are the most commonly used. Specifically, six out of 18 centers employ a 2.5 mm slice thickness, whereas another five out of 18 centers use a 2 mm thickness. Furthermore, five out of 18 centers opt for a finer 1.5 mm slice thickness, and two centers utilize a 3 mm slice thickness for CT scans.

Effective evaluation and management of tumor motion are critical for maintaining accuracy in proton therapy, particularly in PBS therapy, where the dose is delivered sequentially on a spot-by-spot basis [24]. Organ and tumor motion can lead to overshooting or undershooting of the proton beam within the body. Moreover, the interaction between tumor motion and dynamic spot delivery—two independent processes—commonly called the interplay effect, can lead to significant underdosage of the target [25,26], and motion mitigation can reduce the dose to organs at risk for patients with liver tumors [27]. Addressing motion is essential throughout the process, starting from the patient’s CT simulation, during treatment planning, and even throughout the treatment delivery. As shown in Figure 2, the majority of the centers (78%) used 4DCT to evaluate the tumor motion amplitude, 17% used repeated Deep Inspiration Breath Hold (DIBH) scans to assess the residual target motion, and one center (6%) did not specify any motion evaluation process. During CT simulations for liver cancer patients, the objective is to restrict target motion to no more than 5 mm or as minimal as possible [28], especially when delivering PBS. In instances where the motion is slight (a few millimeters) or non-existent, patients can undergo simulations with free breathing (FB). For those requiring motion management, a diverse array of techniques is employed. Among the 18 centers, a total of 35 distinct responses were received regarding motion management strategies, indicating that more than two management strategies can be combined for a single treatment. These include using DIBH techniques (12 out of 35), abdominal compression (11 out of 35), rescanning (10 out of 35), and gating (2 out of 35), whereas no centers used any tumor tracking (0 out of 35), as illustrated in Figure 2. For the DIBH technique, 5 out of 12 centers used the SDX voluntary breath-hold technique (DYN’R Medical Systems, Aix-en-Provence, France) [25], 3 out of 12 used ABC (active breathing control) [29], 3 out of 12 centers used RMP (real-time positioning management, Varian Medical Systems, Palo Alto, CA, USA) [30], and one out of 12 centers used OSMC system (Optical Surface Management System) [31]. Volumetric and in-layer rescanning/repainting are two distinct delivery strategies employed to manage the interplay effect. Among the ten responses received, eight centers utilized the volumetric rescanning method, whereas the remaining two centers opted for the in-layer rescanning method. Of the centers using volumetric rescanning, three reported having systems capable of performing this method automatically, whereas the other five required manual intervention for volumetric rescanning.

### 3.4. Treatment Planning and Quality Assurance (QA)

**Figure 3:**
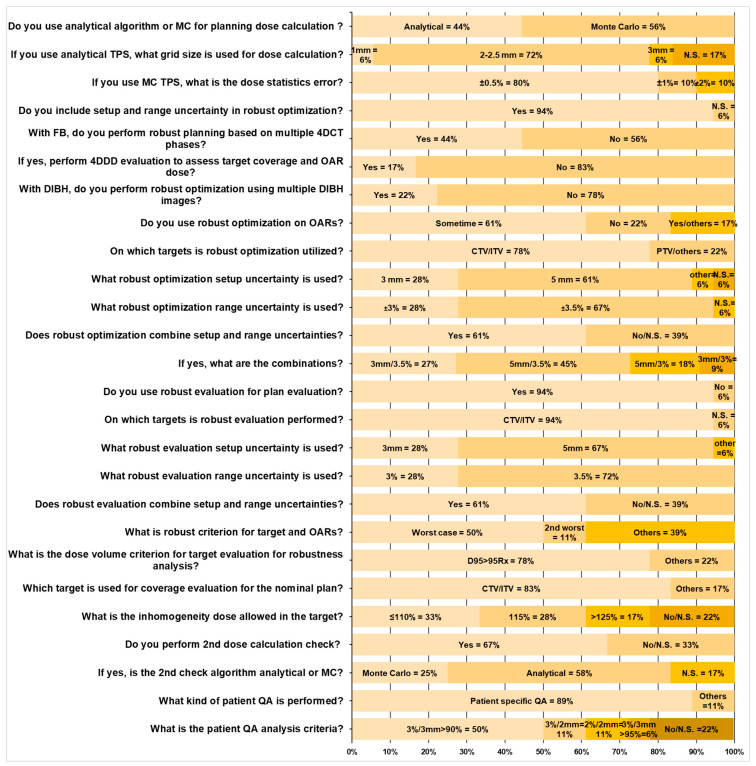
The statistics from the treatment planning and QA survey results for liver SBRT and hypofractionation therapy.

Plan robustness, dose calculation, and delivery accuracy should be assured for proton liver treatment. The patient’s anatomy, setup errors, motion patterns, and amplitude are inevitably different from those of day-to-day treatment. The above perturbations should be considered during the plan optimization and plan evaluations. Figure 3 summarizes the key questions and statistics in treatment planning and QA. The vast majority of centers (17 out of 18) have implemented various strategies to take care of the plan’s robustness. Liver treatment planning also follows these considerations to reduce the dosimetry uncertainties. The most widely used clinical robustness optimization and evaluation method is to assess the dosimetric impact of setup errors and CT calibration uncertainties. As summarized in Figure 3, 11 of the 18 centers used setup errors ±5 mm in all three directions, five centers used ±3 mm setup errors, one center used site-specific setup errors, and one center did not specify any values. Twelve of the 18 centers used ±3.5% CT HU to relative stopping power (RSP) conversion uncertainties, five centers used ±3.0% uncertainty value from clinical practice, and one center did not report their value. Most centers (14 out of 18) used ITV/CTV as the target volume for robust optimization, and the rest (4 out of 18) used PTV or other targets. Besides the robust optimization, 17 of the 18 centers performed robust plan evaluation. With the combination of setup errors and RSP conversion uncertainty, multiple perturbation scenarios can be evaluated on DVH and 3D dose distributions. The doses to target and OARs’ are assessed compared to the nominal treatment plan. As shown in Figure 3, the clinical decision is made based on the worst case (9 out of 18 centers), second-worst case scenario (2 out of 18 centers), or other practical prioritization (7 out of 18 centers) [28,30,32,33,34], such as statistical case scenario method [35]. Among the 18 centers surveyed, 14 centers used the criterion of D95% ≥ 95% of the prescribed dose to evaluate target coverage, whereas the remaining 4 centers assessed target coverage on a case-by-case basis, considering clinical conditions. Target dose inhomogeneity was another critical factor in assessing plan quality. Six of the 18 centers allowed hotspots up to 110% of the prescription dose, 5 permitted hotspots up to 115%, and 3 accepted hotspots up to 125%. The remaining four centers did not specify criteria for dose inhomogeneity within the target for plan evaluation.

Eight out of 18 centers used the analytical dose calculation engine in their TPS for treatment planning, and 11 used the Monte Carlo algorithm. As recommended by Lin et al. [36], the dose calculation accuracy was validated by using an independent dose calculation engine, either a Monte Carlo (3 out of 18 centers) or analytical algorithms (7 out of 18 centers), whichever was available. The other eight centers did not specify if the secondary dose calculation check was implemented in their clinical practice.

A patient-specific quality assurance (QA) program is recommended to validate the treatment accuracy using the 2D ion chamber array [35,36,37,38] or a similar system [39]. Patient QA is essential in identifying the deficiencies of the TPS, especially in some extreme clinical scenarios, such as very small targets. Small volume ion chambers or high spatial resolution detector arrays are highly recommended when the 50–50% dose region is less than 25 mm [26]. Independent dose calculations by a secondary dose calculation engine, providing additional confidence in TPS calculation accuracy, can be used as part of patient-specific QA [40]. The treatment delivery log files record the scanning spots’ position, and MUs can be analyzed to detect the position and delivered dose accuracy, which can be combined with measurement for patient-specific QA purposes [40,41,42]. Among the 18 centers, 16 measured patient-specific QA, whereas two did not specify any QA procedures—the passing criteria utilized gamma comparison methods. Nine centers used a 3 mm/3% criterion, two centers used 2 mm/3%, and two centers used 2 mm/2%, all employing a 90% passing rate to determine pass or failure. Only one center reported using a 3 mm/3% criterion with a tighter passing rate requirement of ≥95%, as shown in Figure 3.

### 3.5. Delivery and IGRT Procedures

The predominant delivery technique among surveyed centers is PBS, with 74% employing this method. Additionally, 22% of the responses (4 out of 18) reported exclusively using the passive scattering technique because these 4 centers are solely equipped with passive scattering capabilities. Image-guided radiation therapy (IGRT) is essential for aligning the tumor and ensuring the accuracy of proton therapy, particularly for treatments involving SBRT or hypofractionation, where fewer fractions are used. IGRT modalities, including kilovoltage (kV) imagers, CBCT, or rail-mounted CT simulator-based image alignment and frequency prescribed by the Radiation Oncologist, were assessed. kV-kV match is primarily used for bony alignment for relatively rigid tumors located in a volume that provides a reasonable alignment and positioning of treatment targets during daily treatment. kV-kV match is also routinely used as an initial alignment before a CBCT is acquired. The radiation oncologist reviews the alignment offline and approves the image alignment if the setup accuracy is within the tolerances.

**Figure 4:**
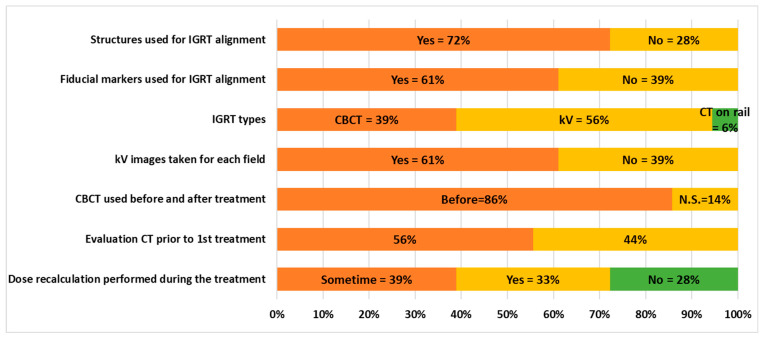
Image-guided radiation therapy used in liver SBRT and hypofractionation treatments.

For proton therapy, three-dimensional (3D) volume anatomy imaging is preferred, providing more details in targets, adjacent OARs, soft tissues, reference ROIs, etc., for more advanced alignments. However, most (10 out of 18) of the proton therapy centers surveyed and delivering liver SBRT or hypofractionation are equipped only with two-dimensional (2D) kV imagers for patient alignment, as shown in Figure 4. Recently constructed proton centers are outfitted with PBS and more advanced onboard CBCT for enhanced imaging capabilities. Seven of the 18 centers have CBCT capabilities, and 86% (6 out of 7) performed CBCT before treatment for alignment. Additionally, one center (6%) is equipped with a rail-mounted CT simulator specifically for IGRT. To enhance the visualization of regions of interest for alignment, 72% of centers (13 out of 18) employ IGRT contours, whereas 28% of centers (5 out of 18) do not use any contours. Due to the low contrast between normal tissue and tumors, 61% of centers (11 out of 18) utilize fiducial markers to improve tumor visibility, whereas the remaining 39% (7 out of 18) do not implement any fiducial markers for target alignment. All proton centers are equipped with kV imagers, and 61% (11 out of 18) prefer to conduct repeated kV imaging between treatment fields to ensure that the patient maintains a consistent position throughout the treatment, whereas the remaining 39% (7 out of 18) do not perform repeated kV imaging between treatment fields.

To assess changes in anatomy, 56% of centers (10 out of 18) conduct repeat QA CT simulations prior to the first treatment, whereas 44% of proton therapy centers (8 out of 18) do not perform any QA CT simulation. Among these, 6 of the 8 centers utilize newly acquired images for dose calculation to evaluate the dosimetric impact resulting from changes in anatomy.

## 4. Discussion

An NRG Oncology survey of United States proton therapy centers found that a majority of respondents have implemented some kind of liver proton SBRT or hypofractionation treatment regimen. However, actual treatment practices, such as prescriptions, treatment planning, motion management, and image guidance, vary among proton therapy centers. These treatment practices are essential to consider when designing clinical trials for liver treatment with proton therapy and can help develop a template for such protocols through the National Clinical Trial Network (NCTN).

Proton therapy provides precise targeting of tumors, minimizing radiation exposure to surrounding healthy liver tissue and other critical structures. This is due to the unique property of protons, which allows them to deposit their maximum energy at the tumor site from Bragg peaks and then stop, reducing the risk of damaging adjacent organs. The ability to concentrate radiation more accurately within the tumor volume allows for higher radiation doses to be delivered to the liver tumor, which could potentially improve treatment effectiveness without increasing side effects in comparison to traditional radiation therapy. Therefore, proton SBRT and hypofractionated therapy are potentially associated with a lower risk of side effects such as RILD and gastrointestinal toxicities, improving the patient’s quality of life during and after treatment. Proton and hypofractionated therapy can be especially beneficial for treating tumors located near critical structures or for patients with underlying liver conditions where sparing as much healthy liver tissue as possible is crucial due to its ready-to-be-combined ultra-high-dose-rate (UHDR) and linear energy transfer optimizations in FLASH radiotherapy [43,44,45,46].

Another critical aspect of proton liver treatment is adaptive strategies. Proton beam ranges are highly sensitive to changes in anatomy and motion amplitude. Repeat CT scans at periodic intervals are an effective method to monitor the anatomy and dose changes. The separation between the CT simulation and the first treatment can be several weeks, providing impetus to perform the initial evaluation scan, which can be ordered to be conducted just before the first fraction of proton therapy. Timely contouring, forward dose calculation, and dose metrics reports based on the new images are needed to identify any significant dosimetric impacts from the change in current conditions. An online CBCT (or onboard CT) device is beneficial for monitoring anatomy changes, like substantial weight loss, to trigger a new CT simulation (adaptive planning). It provides high-quality patient alignment for both target and OARs, while using CBCT images for accurate dose calculation is still challenging due to the large x-ray scatter effect limiting the image quality. In liver SBRT or hypofractionation treatments with fewer fractions, it may not be feasible to start an adaptive plan immediately upon identifying significant anatomical changes during treatment. Therefore, online adaptive planning becomes a potentially more optimal approach to address dosimetric discrepancies. Using a combination of deformable registration tools and artificial intelligence in generating synthetic CT from CBCT has been explored for improved dose calculation for lung and head and neck treatment in studies [47,48,49]. Although high consistency was shown in photon-based studies, it still cannot currently replace CT for planning in proton therapy, and accurate dose assessment should be performed using CT images for liver treatment. Developing online adaptive strategies will benefit liver proton SBRT and hypofractionation treatments.

It was observed that 7 of the 18 centers have onboard CBCT capabilities, and one center is equipped with a CT-on-rails system. The majority of the remaining centers rely solely on 2D kV imaging for patient alignment. Three-dimensional volumetric IGRT is particularly important for high-dose, hypofractionated treatments such as SBRT, as it provides the precision and confidence needed to accurately target complex cases. Ideally, all newly established proton centers should be equipped with 3D volumetric IGRT systems, which would facilitate the broader adoption of proton therapy for liver SBRT and hypofractionated regimens. To ensure high-quality alignment, future clinical trials will likely require the use of 3D volumetric imaging—potentially in combination with fiducial markers—to enhance tumor localization and treatment accuracy.

Another emerging technique in image-guided photon therapy is magnetic resonance (MR)-guided adaptive radiation therapy, which provides superior contrast in soft tissues compared to CBCT image quality [50,51]. This technique has proven effective in several anatomic treatment sites for dose escalation in challenging-to-treat tumors [52,53,54]. Recent developments combining MRI with proton therapy have demonstrated the ability to perform real-time tracking of moving tumors. This advancement could significantly enhance image-guided proton therapy and improve online adaptive therapy [55].

Although proton SBRT represents a significant advancement in liver cancer treatment with its potential for greater precision and fewer side effects, it is essential to consider the challenges associated with cost, accessibility, and the existing evidence base. One of the primary areas of potential growth lies in technological advancements within proton therapy equipment and planning software [56,57]. Innovations such as novel delivery methods using single-energy beams with ultra-high dose rate and more compact and cost-effective proton therapy systems could address current limitations related to the high costs and extensive space requirements of proton therapy facilities [44,45,58], as well as current geospatial disparities in access to proton therapy [59]. Additionally, advancements in real-time imaging and motion management technologies are essential to improve treatment precision, especially for organs subject to motion, like the liver. Enhanced imaging techniques would allow for more accurate tumor targeting, reducing radiation exposure to surrounding healthy tissues and minimizing side effects.

The promising results from a single-arm, phase II, multi-institutional clinical trial and other reports have led to an ongoing phase III NRG Oncology GI003 trial in unresectable, localized HCC, which can help to better understand the role of proton therapy in preserving normal liver functions and improving survival in this patient population [23]. Such a large-scale, randomized, controlled trial comparing proton therapy with conventional radiation therapy is poised to provide the robust data needed to establish its efficacy, safety, and cost-effectiveness more conclusively. Furthermore, research into patient selection criteria will help identify patients most likely to benefit from proton therapy, optimizing treatment outcomes and resource allocation.

## 5. Conclusions

The survey conducted among North American proton treatment centers highlights the growing adoption of proton SBRT and hypofractionated therapy in liver cancer treatment, reflecting an evolving practice pattern. The integration of treatment planning, motion management techniques, and 3D volumetric IGRT further supports the precision required for liver SBRT, pointing towards a future where proton therapy could become a more accessible and effective treatment option for liver cancer. This survey provides insights into the current practices and status, highlighting substantial opportunities to broaden the application of proton SBRT and hypofractionated therapy. These findings can serve as valuable clinical guidance for new proton centers aiming to adopt these therapies for liver cancer patients.

## Figures and Tables

**Table 1:** Proton system vendors, delivery techniques, and treatment planning systems (TPSs) used for liver SBRT and hypofractionated therapy in the US.

Vendors	Count	Rate	Techniques	Count	Rate	TPS	Count	Rate
IBA	8	44%	PBS	14	74%	RayStation	10	56%
Mevion	4	22%	Passive Scatter	4	21%	Eclipse	6	33%
Varian	2	11%	Uniform scanning	1	5%	Others	2	11%
Hitachi	3	17%						
Optivus	1	6%						
Total	18			19 *			18	

* One center uses both PBS and uniform scanning techniques.

## Data Availability

All the data has been shared in this publication.
